# Prominent radiative contributions from multiply-excited states in laser-produced tin plasma for nanolithography

**DOI:** 10.1038/s41467-020-15678-y

**Published:** 2020-05-11

**Authors:** F. Torretti, J. Sheil, R. Schupp, M. M. Basko, M. Bayraktar, R. A. Meijer, S. Witte, W. Ubachs, R. Hoekstra, O. O. Versolato, A. J. Neukirch, J. Colgan

**Affiliations:** 1 Advanced Research Center for Nanolithography, Science Park 106, 1098 XG Amsterdam, The Netherlands; 2 Department of Physics and Astronomy, and LaserLaB, Vrije Universiteit, De Boelelaan 1081, 1081 HV Amsterdam, The Netherlands; 3 Keldysh Institute of Applied Mathematics, Miusskaya Square 4, 125047 Moscow, Russia; 4 Industrial Focus Group XUV Optics, MESA+ Institute for Nanotechnology, University of Twente, Drienerlolaan 5, 7522 NB Enschede, The Netherlands; 5 Zernike Institute for Advanced Materials, University of Groningen, Nijenborgh 4, 9747 AG Groningen, The Netherlands; 6 Los Alamos National Laboratory, Los Alamos, NM 87545 USA

**Keywords:** Electronic structure of atoms and molecules, Laser-produced plasmas

## Abstract

Extreme ultraviolet (EUV) lithography is currently entering high-volume manufacturing to enable the continued miniaturization of semiconductor devices. The required EUV light, at 13.5 nm wavelength, is produced in a hot and dense laser-driven tin plasma. The atomic origins of this light are demonstrably poorly understood. Here we calculate detailed tin opacity spectra using the Los Alamos atomic physics suite ATOMIC and validate these calculations with experimental comparisons. Our key finding is that EUV light largely originates from transitions between multiply-excited states, and not from the singly-excited states decaying to the ground state as is the current paradigm. Moreover, we find that transitions between these multiply-excited states also contribute in the same narrow window around 13.5 nm as those originating from singly-excited states, and this striking property holds over a wide range of charge states. We thus reveal the doubly magic behavior of tin and the origins of the EUV light.

## Abstract (web summary)

Extreme ultraviolet (EUV) light is entering use in nanolithography. Here the authors discuss experimental and theoretical results about the prominent role of multiply-excited states in highly charged tin ions in the mechanism of EUV light emission from laser-produced plasma.

## Introduction

**Fig. 1:**
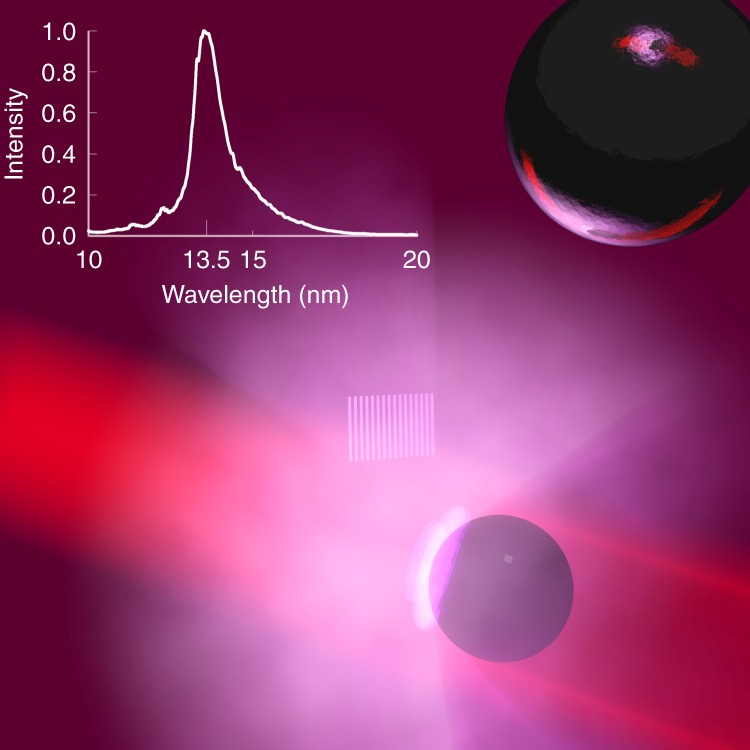
Generation of extreme ultraviolet light. Laser-produced plasma based on the irradiation of tin (Sn) microdroplets by a high-energy ns-pulsed laser. This hot and dense plasma contains highly charged ions to generate extreme ultraviolet (EUV) light near 13.5 nm wavelength relevant for state-of-the-art nanolithography. A transmission grating spectrometer, set up under a −60° angle with respect to the laser light propagation direction, enables unraveling the EUV spectrum. Figure by Tremani/ARCNL.

The complex, exotic electronic structure of highly charged ions of tin (Sn) renders these ions of particular technological value as the enabler of next-generation nanolithography^1–8^. They are employed as emitters of photons in a narrow band closely matching the 2% reflection bandwidth centered at 13.5 nm of the most efficient multilayer optics^9^. This short-wavelength radiation is used to imprint smaller features on commercial microchips. The aptness of Sn ions to this application stems from their open-4*d*-subshell structures^10–20^. Within these structures, Δ*n* = 0 one-electron-excited configurations are very well documented in the literature to decay to the ground state manifold via a multitude of transitions clustered together in unresolved transition arrays (UTAs)^21^, centered in the industrially relevant band around 13.5  nm. Moreover, the average excitation energies of these configurations are similar across the isonuclear sequence Sn^11+^–Sn^14+^, making all these charge states excellent radiators of 13.5-nm light. In industrial applications, Sn ions are bred in laser-produced plasmas (LPPs) driven by a 10-μm-wavelength CO_2_-gas-laser cf. Fig. 1. Incorporation in EUV lithography of solid-state lasers, given the advances in their output power, appears promising. Switching to 1-μm-wavelength Nd:YAG lasers, for example, would be beneficial given the reduction of the required floor area and a strongly improved efficiency of converting electrical power to laser light, reducing carbon footprint. The tenfold decrease in laser wavelength *λ* increases the critical plasma electron density *n*_c_ by two orders of magnitude, *n*_c_ ∝ *λ*^−2^. This higher critical density causes EUV radiation to be created in plasma regions of higher density, and with overall larger optical depth^22^. Significant self-absorption of the emitted radiation in such dense, partially opaque plasma could lead to a broadening of the spectral emission out of the 2% bandwidth of interest, reducing efficiency. In the context of understanding and supporting the drive laser wavelength change in future industrial sources, calculation of complete and accurate opacity spectra and of the atomic data therein is essential for predictive simulations of source performance. These data are needed in radiation hydrodynamics codes^23–26^ and for the calculation of emission spectra. Without such accurate opacity data, the capability for predictive modeling would be severely impaired as, for example, modest underestimations of opacity could lead to significant underestimation of required drive laser intensities. The level of detail in the atomic structure necessary to ensure accurate simulations is an open question. In fact, long-standing discrepancies exist between the measurements of Sn opacity^27^ and various theoretical calculations using a variety of atomic structure and plasma codes^19,27,28^.

This paper identifies the main culprits of the historical discrepancies and addresses them, in order to generate reliable opacity spectra. These spectra are then shown to be in excellent agreement with the emission from a droplet-based EUV source. We find that EUV light predominantly originates from transitions between multiply-excited states. Contrary to the prevailing view, contributions from one-electron-excited states are minor. Moreover, we find that transitions between these multiply-excited states also strongly contribute to the same narrow 2% bandwidth around 13.5 nm as those originating from the well-known singly-excited states. This serendipitous alignment of transitions furthermore occurs over a range of charge states Sn^11+^–Sn^14+^. A doubly magic behavior of tin is revealed. Having uncovered the true origins of the EUV light, our calculations will thus enable predictive modeling of future, more powerful and efficient laser-driven plasma sources of EUV light.

## Results

### Level structure of tin ions

**Fig. 2:**
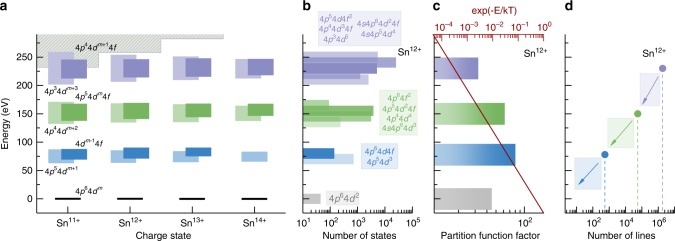
Energy levels of tin ions and their population. **a** Schematic energy level diagram of the ions Sn^11+^–Sn^14+^, showing only selected Δ*n* = 0 transitions for clarity. The ground-state configurations of these ions take the form 4*d*^*m*^, with *m* = 3 − 0. The lowest-lying level of each ground-state manifold is shown in black and is fixed at an energy of 0 eV. The energy "spread'' of an excited configuration is illustrated by a rectangle, centered at the average energy of the configuration and whose width represents the first moment of the level distributions. The shaded gray area denotes the ionization potentials of the ions. For the Sn^12+^ ion example case: **b** Statistical weight ${\sum }_{J}(2J+1)$ per configuration. **c** Partition function factor, defined as ${\sum }_{i}(\exp (-{E}_{i}/kT){\sum }_{J}(2J+1))$ in configurations *i* grouped by excitation degree and (in red) exponential Maxwell–Boltzmann weighting factor $\exp (-E/kT)$ with *T* = 32 eV. **d** Number of lines per grouped configuration. In the final calculations, more excitations from further Δ*n* = 0 permutations and excitations into the *n* = 5 shell are also considered, resulting in a total of 10^10^ dipole allowed transitions.

The electronic energy level scheme presented in Fig. 2 exemplifies the characteristics in the atomic structure that need to be captured to accurately model a Sn plasma for nanolithograpic applications. This structure shows the average energies and widths of some of the typical configurations that play a role in the generation of EUV photons. The most notable phenomenon is that the excitation energies of electrons within the *n* = 4 manifold shown are rather independent of the occupation of the manifold itself: the energy required to promote a 4*p* electron to the 4*d* subshell is almost the same regardless of the number of electrons in any of the other subshells. Fig. 2a shows that this holds either when changing charge state or excitation degree. This remarkable fact notwithstanding, the predominant view has been that only transitions of type 4*p*^6^ 4*d*^*m*^ − 4*p*^6^ 4*d*^*m*−1^4*f*  + 4*p*^5^ 4*d*^*m*+1^ (with *m* = 3. . . 0 for *q* = 11. . . 14 in Sn^*q*+^), that is transitions from the singly-excited configurations significantly contribute to the emission of EUV photons. We will demonstrate the contrary: the contributions from multiply-excited states dominate. Taking as an example the Sn^12+^ ion, Fig. 2b shows that the multiply-excited configurations, due to their large number of levels and high statistical weights (Fig. 2c), have large populations despite the lower excitation probabilities that are here described using a Boltzmann distribution. Surprisingly we find that, for example, the triply-excited states have similar partition function contributions as the singly-excited states. These configurations therefore also must make an equally significant contribution to the production of EUV photons. The large number of decay channels is exemplified in Fig. 2d with configurations decaying via electric dipole transitions towards the lower levels, which in turn decay again radiating similar energy photons. We note that Fig. 2 shows only a relatively small example of the number of configurations produced by successive Δ*n* = 0 excitations from the ground configuration. In the full calculations, presented in the next section, further Δ*n* = 0 excitations, and excitations into the *n* = 5 shell, are also included. Many of these transitions also make significant contributions to the emission in the relevant wavelength range.

In the following, we present the opacity spectrum of a Sn plasma calculated in local thermodynamic equilibrium (LTE) at conditions relevant for the production of EUV light in an industrial setting. The calculations were performed using the Los Alamos code ATOMIC^29,30^, which takes as input state-of-the-art atomic data calculated with the Los Alamos suite of atomic codes^31,32^. The atomic structure was calculated using the semi-relativistic Hartree-Fock approach implemented in the CATS code (based on Cowan’s code^33^). These data are used by ATOMIC to calculate opacity spectra under the assumption of LTE with input from equation-of-state calculations performed with ChemEOS^34,35^, which ensures convergence of the partition function and thermodynamic consistency.

### Atomic structure calculations

One of the most challenging aspects in calculations of Sn plasma opacities is the atomic structure of the highly charged Sn ions, due to their open 4*d*-subshells and the existence of strong configuration-interaction (CI) between levels in the *n* = 4 manifold. The difficulties associated with accurate calculations of these level structures were presented in a recent study of Colgan et al. whereby Sn opacities, calculated using the aforementioned Los Alamos codes, were shown to be sensitive to the number of configurations included in the CI expansion^19^. However, without a suitable experimental benchmark, it was not possible to determine whether sufficient configurations were included in the atomic structure models.

In the current work, to ensure that the position of multiply-excited levels and their oscillator strengths are calculated to the highest possible accuracy, full CI effects are taken into account for most of the single, double, and triple excitations of valence, 4*s*, and 4*p* electrons of the ground-state configuration into the majority of *n* = 4 and *n* = 5 subshells. The list of configurations that were included in the full CI calculation for Sn^12+^ is presented in Table 1. This list comprises 94 configurations and generates over 3 × 10^5^ fine-structure levels and more than 10^10^ dipole-allowed transitions, far more than were taken into account in other modeling work (see, for example, the work of Sasaki et al.^36^ on CO_2_-laser-driven Sn plasma). Similar sets of configurations were used for the neighboring ion stages. Moreover, we also included a significant number of other configurations for which the transitions were included using intermediate-coupling^19^. This mixed approach, called ‘2-mode’, maintains the accuracy of CI calculations for the most important transitions in the EUV regime, while retaining other levels that represent more highly excited states that are necessary for an accurate partition function and opacity at higher photon energies. Thus our approach meets the twin requirements of accuracy (for the crucial transitions), and completeness (for partition function convergence).

**Table 1:** Configuration list.

Inner subshells		Outer subshells
4*s*^2^4*p*^6^	+	{4*d*^2^, 4*d*4*f*, 4*f*^2^, 4*d*5*l*, 4*f*5*l*}
4*s*^2^4*p*^5^	+	{4*d*^3^, 4*d*^2^4*f*, 4*d*4*f*^2^, 4*d*^2^5*l*, 4*d*4*f*5*l*}
4*s*^2^4*p*^4^	+	{4*d*^4^, 4*d*^3^4*f*, 4*d*^2^4*f*^2^, 4*d*^3^5*l*, 4*d*^2^4*f*5*l*}
4*s*^1^4*p*^6^	+	{4*d*^3^, 4*d*^2^4*f*, 4*d*4*f*^2^, 4*d*^2^5*l*, 4*d*4*f*5*l*}
4*s*^2^4*p*^3^	+	{4*d*^5^, 4*d*^4^4*f*, 4*d*^3^4*f*^2^, 4*d*^4^5*l*, 4*d*^3^4*f*5*l*}
4*s*^1^4*p*^5^	+	{4*d*^4^, 4*d*^3^4*f*, 4*d*^2^4*f*^2^, 4*d*^3^5*l*, 4*d*^2^4*f*5*l*}
4*s*^2^4*p*^6^	+	{5*s*^2^, 5*s*5*l*, 5*p*^2^, 5*p*5*l*, 5*d*^2^, 5*d*5*l*, 5*f*5*g*}
4*s*^2^4*p*^5^4*d*	+	{5*s*^2^, 5*s*5*p*, 5*s*5*d*}

List pertains to the configurations included in the full configuration interaction (CI) calculation for Sn^12+^ as used in the main text. Here, angular momenta *l* = 0–4 → *s*–*g*.

The list of configurations adopted for a given ion stage was determined by systematically increasing the number of configurations allowed to interact, and identifying for which configuration sets the positions of the dominant transitions converge. It is well-known that ab initio calculations performed in this manner do not necessarily reproduce experimental spectra to a high degree of accuracy. To circumvent this, it is standard practice in Cowan code calculations to introduce so-called scaling factors which pre-multiply the radial integrals appearing in the Hamiltonian matrix elements. As noted by Cowan^33^, these scaling factors account for the ‘infinity of small perturbations’ that are necessarily omitted in practical atomic structure calculations. Normally, a reduction of 10–15% of the radial integrals, that is, applying scaling factors of 0.85–0.9, can bring theoretical calculations of level energies (and subsequently calculated transition wavelengths) into very good agreement with experimental observations. In our CATS calculations, the scaling factor is set to a standard 0.87 based on previous CATS calculations performed on a wide range of elements and charge states. When using the configuration set in Table 1, already much larger than previous calculations presented in ref. ^19^, the position of the major transitions to the ground-state configuration (for example, $4{d^{2^1}}$G_4_ → 4*d*4*f* ^1^H_5_ in Sn^12+^) are in excellent agreement with the experimental observations in ref. ^14^. Calculations were then performed using ATOMIC including atomic structure data for all relevant ion stages calculated in a manner similar to Sn^12+^. These calculations produced an intense emission feature in good qualitative agreement with the measured spectrum, apart from a crucial shift in the central position of the emission feature towards shorter wavelength. In fact, the feature was positioned outside the relevant 2% emission band for nanolithography. Since it was established that the well-known transitions to the ground manifold are correctly calculated, the discrepancy must originate from inaccurate positioning of transitions between excited states.

This unexpected finding led us to re-consider our atomic structure calculations for the excited-excited transitions. Little data for such excited transitions are available for the Sn^11+^–Sn^14+^ ions. For Sn^14+^, the calculated electronic structure differed from the interpretation of charge-exchange measurements (and accompanying calculations) of D’Arcy et al.^37^ by around 2%  in wavelength position. We found that reducing the scaling factors in CATS to 0.75 yielded much better agreement with these data and with the experimental charge-exchange emission spectra of Ohashi et al.^17^. This further reduction may account for greater correlation effects between these high-energy configurations, arising from their high density of states.

Adopting scaling factors of 0.87 and 0.75 for the transitions to the ground manifolds and for transitions between excited states, respectively, opacity spectra are calculated at the representative temperature and density of 32 eV and 0.002 g cm^−3^ (~10^20^ e^−^ cm^−3^). These plasma conditions, used throughout this paper, are typical for a 1-μm-driven LPP tailored for emission of 13.5-nm photons, as suggested by radiation hydrodynamic simulations^24,25^ (see “Methods” section). Light emission is described as occurring at sub-critical density, close to the sonic surface of the ablation front at an electron density of several 10^20^ e^−^ cm^−3^. These density and temperature values also support the LTE approach adopted (see Methods).

**Fig. 3:**
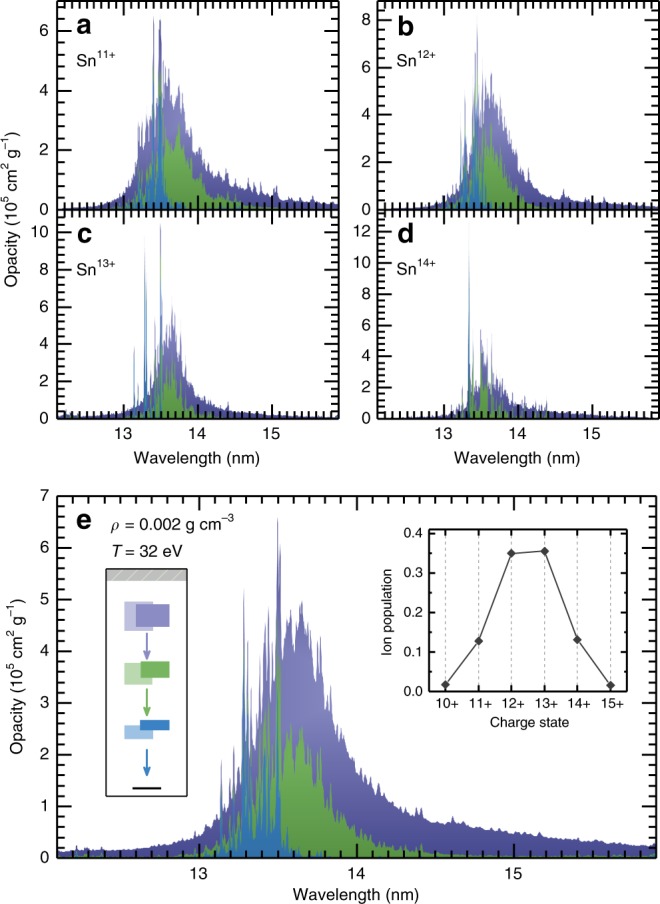
Opacity spectra of tin ions. Calculations were performed for a 32 eV, 0.002 g cm^−3^ Sn plasma in local thermodynamic equilibrium. The shaded areas represent the cumulative contributions (that is the next contribution is plotted stacked on top of the previous one) stemming from different types of excited states. They are divided according to the energy of the lower state into which the ions radiatively decay: in blue, transitions into the ground state manifold (from single-electron excited states); in green, transitions into levels with energies between 0 and 150  eV (comprising mainly transitions between singly- and doubly-excited states); in purple, transitions occurring between doubly-excited states (lying above 150  eV) and higher-lying multiply-excited states. **a**–**d** Opacity spectra of the individual Sn ions. **e** Total opacity spectrum. The left inset contains the simplified atomic structure of Sn^13+^ (see Fig. 2 and main text), while the right inset shows the relative charge state population of the plasma. All spectra are convoluted with a Gaussian profile to improve the visibility of the various contributions.

In Fig. 3, the contribution of the four ion stages Sn^11+^–Sn^14+^ to the total opacity is shown. Each spectrum shows the three major bound-bound contributions, which can be loosely associated with singly-excited, doubly-excited, and triply-excited states (see Fig. 2). Remarkably, for this choice of temperature and density, the well-known transitions to the ground levels comprise only 11% of the total opacity in the 5–20 nm range. The remaining 89% is associated with higher-lying transitions: 26% is attributed to transitions between singly- and doubly-excited states, 25% to transitions between doubly- and triply-excited states, and 38% is associated with higher excitations. Even when considering the opacity in a 2% bandwidth around 13.5 nm, the transitions from singly-excited configurations only account for 19% of the total opacity.

### Comparison to experiment

In order to benchmark our present calculations, we have made comparisons with experimental laser-produced tin-plasma spectra recorded for a variety of laser intensities, which determine the plasma properties such as temperature and degree of ionization^38^. Guided by our radiation-hydrodynamics simulations (see below), we performed ATOMIC calculations at predicted ranges of plasma temperatures and densities, and compared these to the measured spectra to ascertain the specific sets of conditions that lead to best agreement between modeling and experiment. The experimental spectra were obtained by irradiating a molten Sn microdroplet, 30 μm in diameter, with a 15-ns-long Nd:YAG laser pulse having a flat-top spatial profile of 96 μm diameter^38,39^. The emission in the EUV regime is recorded using a wavelength-calibrated spectrometer^40^. The experiment is described in further detail in ref. ^38^. Spectra have been recorded at three distinct laser intensities: 1.4 × 10^11^  W cm^−2^ (this value gives optimal performance with respect to EUV emission^38^), 6.6 × 10^10^ W cm^−2^, and 3.9 × 10^10^ W cm^−2^.

To enable a comparison between opacity calculations and experimental emission spectra, we must adopt a model for radiation transport through the plasma medium. In LTE, the complexity of the radiation transfer problem is reduced since the (spectral) emissivity *η*_*λ*_ and the (spectral) opacity *κ*_*λ*_ are linked by the relation *η*_*λ*_ = *B*_*λ*_ ⋅ *κ*_*λ*_ ⋅ *ρ*^41^, where *B*_*λ*_ is Planck’s spectral radiance and *ρ* the mass density (the product of *κ*_*λ*_ and *ρ* being the absorptivity *α*_*λ*_). Generally, even in the 1D approximation, the radiation transport equation should be solved numerically along the plasma column leading to the observer. This would necessitate the calculation of opacities for each (*ρ*,*T*) pair. Such an endeavor, particularly in view of the level of detail in the opacity calculations here presented, is beyond the scope of this paper. Instead, a single-temperature, single-density approach is here employed. Indeed, recent experiments by Schupp et al.^22^ have indicated that a dominant fraction of the EUV emission may be produced in such a quasi-stationary^24^ single-density, single-temperature region. For such a medium, the spectral flux *I*_*λ*_ can be determined using the simple solution ${I}_{\lambda }={B}_{\lambda }\left[1-\exp (-\tau )\right]$, with the optical depth *τ* defined as the product between *α*_*λ*_ and the transport path-length *L*. The temperature of the opacities are chosen such that the calculated charge state contributions matched the observed one. In order to justify the choice of density and path length, we have undertaken radiation hydrodynamic simulations using the RALEF-2D code^24,25,42^. These simulations indicate that the vast majority of the emission originates in a 10- to 30-μm-thick plasma having density on the order of 10^20^ e^−^ cm^−3^, rather independent of laser intensity (see Methods section). In our comparisons below, we use a constant 30-μm path length at 10^20^ e^−^ cm^−3^ density.

**Fig. 4:**
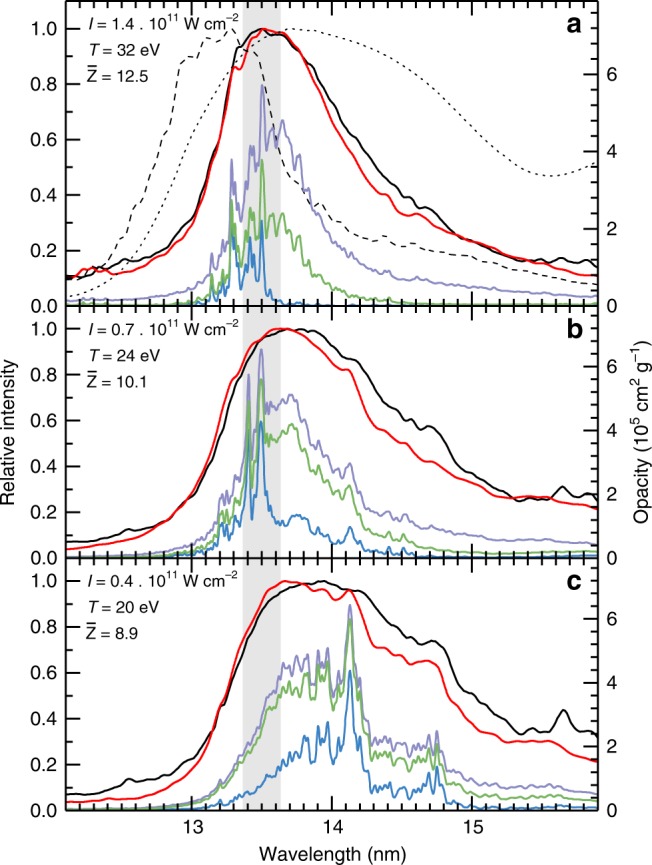
Comparing calculation to experiment. Experimental spectra (black solid lines) and calculated fluxes (red solid lines) are shown, normalized to their respective maximum. The spectral fluxes are the result of the 1D radiation transport through a single-density (0.002 g cm^−3^), single-temperature plasma (see main text). **a** Spectral flux calculated using an opacity spectrum from ref. ^19^ (dashed line) and the spectral flux obtained from HULLAC calculations^27^ (dotted line). Opacity spectra, broken down according to the various contributions illustrated in Fig. 3 are also shown. The mean charge state $\bar{\,\text{Z}\,}$ of the calculation is given as well. The shaded gray area highlights the industrially-relevant 2 % bandwidth around 13.5 nm. **b**, **c** Same as in **a** but for two lower laser intensities and associated plasma temperatures.

In Fig. 4, a comparison between the experimental emission spectra and the spectral fluxes obtained from applying the aforementioned 1D radiation transport model to the ATOMIC opacity calculations is presented for three different laser intensities. Overall, the level of agreement is excellent. Fig. 4a shows the spectrum for the laser intensity 1.4 × 10^11^ W cm^−2^. The spectral flux calculated using the single-density, single-temperature approach is able to reproduce the experimental emission strikingly well. The figure also shows the plasma opacity from Fig. 3, which makes apparent that without the contributions from the multiply excited states it would not be possible to fully explain the experimental spectrum. To further highlight the importance of these transitions, our results are compared with calculations from previous works. The dashed line was obtained using the opacity from Colgan et al.^19^, which, as discussed in a previous section, perfectly exemplifies the shift of the main emission feature towards shorter wavelengths, arising from inaccuracies in the calculated line positions for transitions between multiply-excited states. The dotted line is based on opacity data from ref. ^27^, generated with the HULLAC code at an electron temperature of 30 eV but at a higher mass density of 0.01 g cm^−3^. To enable the comparison at similar optical depth, the path length used to calculate the spectral flux was five times shorter. These calculations significantly overestimate the width of the main emission feature and are in poor agreement with the experimental spectra and, while some disparities could be explained by the density difference, the overall discrepancy may be attributed to the atomic structure employed.

Figure 4b and c show the comparison between calculations and experiment at lower laser intensities. These spectra clearly exhibit the spectral signature of lower Sn charge states. The data are still in good agreement, even though some deviations are observed in the 14–16 nm region. Indeed, our assumptions might (partially) break down at lower intensities, due to inapplicability of the single-density, single-temperature approach or deviation from the calculated charge state balance. The opacity breakdowns for these two cases show that the relevance of the multiply-excited states decreases for the lower plasma temperatures but they are still necessary for complete opacity spectra.

## Discussion

It is interesting to consider why multiply-excited states appear to be so important in Sn plasma. Lower Z elements under similar conditions, for example, Al or Fe, are also ionized approximately ten times; however, this results in ion stages with much simpler configurations, such as open-2*p*- or open-3*p*-subshells in Al and Fe respectively. Multiply-excited states in these subshells have much smaller statistical weights compared to the multiply-excited states of Sn, and so their relative contribution to the plasma emission is also much smaller. If instead one looks once again at open-4*d*-subshells but in a lighter element, such as neutral Sr, the multiply-excited states are energetically much further away from the ground state due to the smaller nuclear charge. Compared to Sn, this significantly reduces their contribution to the partition function. For example, the 4*p*^5^ 4*d*^3^ configuration in neutral Sr at a temperature of 1 eV contributes 10^−13^ times less than in the case of the same configuration for Sn ions in a 30 eV plasma. For all of these reasons, Sn finds itself in this peculiar position in which, due to the plasma conditions necessary for nanolithography, the complicated structures of these multiple, *n* = 4 excited-electron configurations play a staggeringly important role. These multiply-excited states will also play an important role in other short-wavelength applications^43^ ranging from beyond-EUV lithography^2^ to water-window imaging^44^ where candidate elements for LPP exhibit strong *n* = 4 − 4 transition arrays that contribute to radiation, with emission wavelength decreasing with the atomic number following a quasi-Moseley law^45^.

In conclusion, we show that, contrary to the prevailing view, the opacity of high-density Sn plasma of relevance for nanolithographic applications are characterized by a remarkably large contribution from multiply-excited states. Multiple electron excitation into the 4*d* or 4*f* subshells leads to states with very high angular momenta and large statistical weights. These configurations are heavily affected by configuration-interaction, making them challenging to calculate accurately. Crucially, the dominant bound-bound transitions involving these multiply-excited states are clustered close to 13.5 nm wavelength, as are the transitions from singly-excited states. These insights finally enable explaining the intense emission feature from Sn LPPs that are right now entering high-volume manufacturing in the nanolithographic industry for the continued progress of miniaturization of semiconductor devices. The calculations are shown to be in excellent agreement with experimental emission spectra from a droplet-based, Sn laser-produced-plasma source of EUV light. Our results will enable accurate simulation of emission spectra from radiation-hydrodynamic simulations of high-density Sn plasmas aiding the development of future more powerful and energy-efficient EUV light sources.

## Methods

### Los Alamos atomic codes

The atomic structure calculations used in our modeling are discussed in detail in the main text. These calculations are augmented by photoionization cross sections computed from the Los Alamos GIPPER code^32^. These data are read into the plasma modeling code ATOMIC. In its LTE mode, ATOMIC computes the partition function for a given temperature & density using the CHEMEOS^34,35^ option, which computes equation-of-state quantities in a chemical picture. ATOMIC then computes the resulting plasma opacity or emissivity using this partition function, coupled with the detailed atomic transition data from CATS.

### Validity of local thermodynamic equilibrium

The large number of available (~10^10^) transitions computationally precludes performing full-detail collisional radiative modeling. It is common to invoke local thermodynamic equilibrium (LTE) in such cases to enable the prediction of experimental spectra using Boltzmann-distributed excitation-population densities. Several validity criteria for LTE are available, mostly revised versions of the original proposed by Griem^46^, for atomic systems of limited complexity. These validity criteria would indicate establishment of LTE at a density scale of 10^20^–10^21^ e^−^ cm^−3^, supporting its invocation in the current work.

**Fig. 5:**
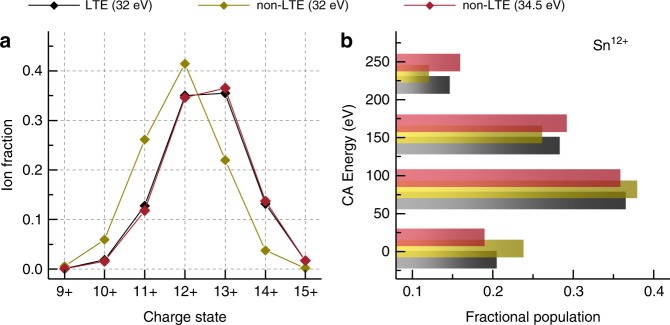
Validity of local thermodynamic equilibrium (LTE). **a** Charge state distribution calculations for a 32 eV, 0.002 g cm^−3^ (~1 × 10^20^ e^−^ cm^−3^) plasma under both LTE and non-LTE conditions; a non-LTE calculation for a 34.5 eV temperature is additionally shown. **b** Fractional population in configurations grouped by excitation degree (as in Fig. 2) under conditions indicated in **a** from configuration-averaged (CA) calculations (see main text). CA energies are offset for better visibility.

To enable a more direct validation, configuration-averaged non-local thermodynamic equilibrium (non-LTE) calculations were also run using ATOMIC in its non-LTE mode. The configuration-average atomic data needed for the non-LTE calculations were generated using the LANL suite of atomic physics codes. The CATS calculations, in this case, were made with the default Cowan scale parameters. Rates of collisional excitation and ionization, and autoionization (and the inverse of these processes) are needed to perform a non-LTE calculation. The generation of these data, plus the need for a full matrix solve of the collisional-radiative problem, means that we normally require about three orders of magnitude more computing resources than a LTE calculation that uses the same number of atomic states. This is why we considered the much simplified configuration-average problem with a small number of configurations, taking into account only Δ*n* = 0, 1 excitations but including the doubly and triply excitations within the *n* = 4 manifold. The calculations, shown in Fig. 5, were performed for a 32 eV, 0.002 g cm^−3^ (~1 × 10^20^ e^−^ cm^−3^) plasma under both LTE and non-LTE conditions. It is immediately apparent that there is difference in the charge state distribution and the obtained non-LTE spectrum would not be relevant for the comparison at the given temperature. Still, a large fraction of the population is shown to be retained in the multiply-excited configurations. For a fairer comparison, following the established approach in Sasaki et al.^36^ (also see the review by Bauche et al.^47^) we slightly increase the non-LTE temperature to 34.5 eV such that the mean charge state $\bar{\,\text{Z}\,}$ in non-LTE equals that of the 32 eV-LTE calculations. This small step in temperature renders the charge state distribution indistinguishable from the LTE one (see Fig. 5). The small change in the temperature needed to reproduce the LTE calculations furthermore strongly indicates that the system is close to LTE at 32 eV and 0.002 g cm^−3^. Photo-excitation by the radiation field, not included in our non-LTE calculations, would bring the system even closer to LTE. At these temperatures, our non-LTE calculations would indicate similarly strong contributions as in LTE of the multiply-excited states to the emission of EUV light from tin LPP.

### Radiation-hydrodynamics simulations

We have performed RALEF-2D simulations to explore where the extreme ultraviolet light is generated in a laser-produced-plasma resulting from the irradiation of a Sn microdroplet by a high-intensity, 1-μm-wavelength laser-pulse. RALEF-2D is a two-dimensional numerical code which solves the 2D single-fluid, one-temperature hydrodynamics equations and the spectral radiation transfer equation, using opacity tables generated with the THERMOS code^48^. In the RALEF-2D code, energy transport via radiation is coupled directly to the fluid through the fluid energy equation, and therefore spectral radiation transport is treated in a self-consistent manner. As has been demonstrated previously, the radiation-hydrodynamic approach implemented in RALEF-2D makes it very apt in simulating systems in which energy transport by thermal radiation plays a significant role^24,25,42,49^. The code was also recently validated against measurements of laser-induced propulsion of Sn microdroplets^50^.

The simulations begin by setting the initial conditions of the system: droplet size, spatial and temporal laser profiles, and laser energy. For the three spectra shown in Fig. 4, the parameters are as follows: 30 μm droplet diameter; box-shaped laser profiles, 96 μm spatially, and 15 ns temporally; laser energies of 170 mJ, 78 mJ, and 47 mJ, corresponding to intensities of 1.4 × 10^11^ W cm^−2^, 0.7 × 10^11^ W cm^−2^, and 0.4 × 10^11^ W cm^−2^. From the simulation results, it is possible to obtain two-dimensional spatial profiles of temperature and density as a function of time. To simplify the analysis, we have generated one-dimensional profiles of these two quantities along a −60° line-out with respect to the laser propagation direction, effectively emulating the observation angle of the spectrometer in the experiment. The duration of the laser beam is sufficient to have a steady-state ablation front, and therefore in the following we will look at the time instant at the end of the laser pulse (before it is turned off).

**Fig. 6:**
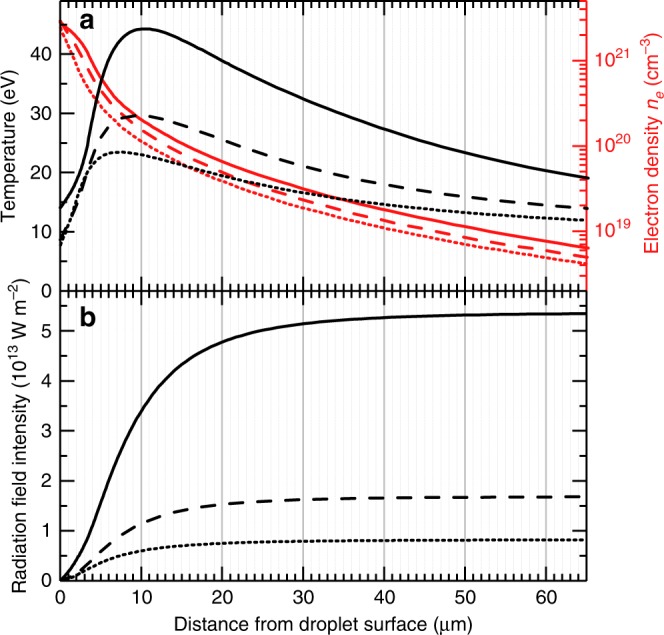
Simulation results. **a** Temperature and density profiles calculated by RALEF-2D simulations for laser intensities of 0.4 (dotted), 0.7 (dashed) and 1.4 (solid) × 10^11^ W cm^−2^, respectively. **b** Radiation field intensity obtained by solving the simplified frequency-integrated transport equation, from Eq. (4), along the density and temperature profiles in **a**.

In Fig. 6a, we plot the spatial variation of temperature and density obtained from the RALEF-2D code along the aforementioned −60° line-out for the three relevant laser power densities. The spatial variation of these two quantities sets the radiation properties of the plasma medium. In order to pinpoint the origin of the intense EUV emission in the simulated plasma, we have undertaken a post-simulation analytic study of radiation transport using these temperature and density profiles. In our simplified approach, we first assume that the effects of scattering are small relative to absorption mechanisms; secondly, we consider frequency-integrated variables to simplify the amount of data necessary for these calculations. In this static limit, all variables are ultimately a function of only the position along the transport length *s* and therefore the radiation transport equation reads:

1 $$\frac{\partial I}{\partial s}=\alpha [B(T)-I],$$

with *I* the frequency-integrated radiation intensity, *s* the path length variable, *α* the non-linearly averaged absorption coefficient, and *B*(*T*) = *σT*^4^/*π*. In order to solve the previous equation, one needs an approximate value for *α*. In general, this quantity is equated to the Planck mean opacity:

2 $$\alpha \approx {\alpha }_{P}\equiv \frac{1}{B(T)} \int_{0}^{\infty }{\alpha }_{\nu }{B}_{\nu }\,\text{d}\,\nu .$$

The Planck mean opacity, in the case of Sn plasma, can be calculated as follows^24^:

3 $${\alpha }_{P}\ [\,\text{m}{}^{-1}\text{}\,]=3.3\cdot 1{0}^{-7}\cdot \rho \ [\,\text{g}\,\text{cm}{}^{-3}\text{}\,]\cdot {T}^{-1}\ [\,\text{eV}\,].$$

This equation allows for the calculation of the spatial variation of *α*_*P*_, denoted *α*(*s*) in the following, using the temperature and density profiles shown in Fig. 6a. Although this approach is indeed a simplified version of that taken in the RALEF-2D code (which explicitly employs density- and temperature-dependent spectral absorption coefficients *α*_*ν*_(*ρ*, *T*) calculated using the THERMOS code), it can be used to identify the position and extent of the EUV-emissive zone. The solution to Eq. (1) reads:

4 $$I(s)=	 \,\,{I}_{0}\exp \left[-\int_{s0}^{s}\alpha (s^{\prime} )\,\text{d}\,s^{\prime} \right]\\ 	+\int_{s0}^{s}\alpha (s^{\prime} )B(s^{\prime} )\exp \left[-\int_{s^{\prime} }^{s}\alpha (s^{\prime\prime} )\,\text{d}s^{\prime\prime} \right]\text{d}\,s^{\prime} ,$$

which can be easily solved numerically using the line-out profiles obtained from RALEF-2D.

The solution to *I*(*s*) is presented, alongside the temperature and density profiles, for the three laser intensities in Fig. 6. These profiles, besides their absolute values, are rather independent of laser intensity. The laser light is found to be dominantly absorbed in the underdense corona before reaching the critical surface, in line with the findings in ref. ^24^. The profiles in panel b clearly show that the vast majority of the radiation field intensity builds up in the first 20 μm, then levelling off as the lower temperature, rarefied plasma does not contribute strongly to the radiation field, neither in emission nor in absorption. In all three cases explored, half of the far-field radiation field intensity is shown (see Fig. 6b) to be achieved at an electron density of 3 × 10^20^ cm^−3^ and a 80% fraction of the final radiation field intensity is built up over a 10 μm path length. These values found are in good agreement with the ones chosen in the 1D radiation model necessary to compare the opacity calculations to the experimental emission spectra, where a path length of 30 μm together with the density of 10^20^ cm^−3^ gives very good agreement with the experimental data. At an electron density of 3 × 10^20^ cm^−3^, the plasma is even closer to LTE and the slightly higher temperature, cf. Fig. 6, at this higher density will result in even larger population fractions in the multiply-excited states given their exponential dependence on the plasma temperature.

On the other hand, temperature peak values given by the code are higher than expected. These results are rather inconsistent with our spectroscopic measurements simply on the basis of charge state balance. If we look at the highest intensity case in Fig. 6, temperatures over 45 eV are observed. At this temperature, we would expect a plasma average charge state above 14+ according to ref. ^24^. This is demonstrably not the case. These discrepancies could be originating from the opacity tables employed in RALEF-2D, which do not include the contribution from multiply-excited states as outlined in the present work, and underline the importance of obtaining accurate opacity tables. Some ambiguity about the plasma temperature which best matches the data remains. As the laser intensities and associated temperatures are shown to have a minor influence on density and length scale results (cf. Fig. 6), these minor inconsistencies do not impact the results of said density and length scales.

## Data Availability

The data that support the findings of this study are available from the corresponding authors upon reasonable request.

## Code availability

RALEF-2D is available upon reasonable request from M.M. Basko. Correspondence and requests for ATOMIC and related codes used in the paper should be addressed to J. Colgan.

## References

[1] Versolato OO (2019). Physics of laser-driven tin plasma sources of EUV radiation for nanolithography. Plasma Sources Sci. Technol..

[2] Tallents G, Wagenaars E, Pert G (2010). Optical lithography: lithography at EUV wavelengths. Nat. Photonics.

[3] Wagner C, Harned N (2010). EUV lithography: lithography gets extreme. Nat. Photonics.

[4] Waldrop MM (2016). The chips are down for Moore’s law. Nat. News.

[5] Banine VY, Koshelev KN, Swinkels GHPM (2011). Physical processes in EUV sources for microlithography. J. Phys. D: Appl. Phys..

[6] Fomenkov I (2017). Light sources for high-volume manufacturing EUV lithography: technology, performance, and power scaling. Adv. Opt. Techn..

[7] Kawasuji Y (2017). Key components technology update of the 250W high-power LPP-EUV light source. SPIE Adv. Lithogr..

[8] Purvis, M. et al. In *Extreme Ultraviolet (EUV) Lithography IX*, vol. 10583, 476—485 (International Society for Optics and Photonics, 2018)

[9] Huang Q (2017). Spectral tailoring of nanoscale EUV and soft x-ray multilayer optics. Appl. Phys. Rev..

[10] Azarov VI, Joshi YN (1993). Analysis of the 4d^7^- 4d^6^ 5p transition array of the eighth spectrum of tin: Sn VIII. J. Phys. B: . Mol. Opt. Phys..

[11] Svendsen W, O’Sullivan G (1994). Statistics and characteristics of xuv transition arrays from laser-produced plasmas of the elements tin through iodine. Phys. Rev. A.

[12] Churilov SS, Ryabtsev AN (2006). Analyses of the Sn IX-Sn XII spectra in the EUV region. Phys. Scr..

[13] Churilov SS, Ryabtsev AN (2006). Analysis of the 4p^6^4d^7^ − (4p^6^ 4d^6^ 4f + 4p^5^ 4d^8^) transitions in the Sn VIII spectrum. Opt. Spectrosc..

[14] Churilov SS, Ryabtsev AN (2006). Analysis of the spectra of In XII-XIV and Sn XIII-XV in the far-VUV region. Opt. Spectrosc..

[15] Ryabtsev AN, Kononov ÉY, Churilov SS (2008). Spectra of rubidium-like Pd X-Sn XIV ions. Opt. Spectrosc..

[16] Tolstikhina, I. Y., Churilov, S. S., Ryabtsev, A. N. & Koshelev, K. N. In *EUV Sources for Lithography* (ed. Bakshi, V.) chap. 4, 113–148 (SPIE Press, 2006).

[17] Ohashi H (2010). EUV emission spectra in collisions of multiply charged Sn ions with He and Xe. J. Phys. B: . Mol. Opt. Phys..

[18] Windberger A (2016). Analysis of the fine structure of $${{\rm{Sn}}}^{11+}-{{\rm{Sn}}}^{14+}$$ ions by optical spectroscopy in an electron-beam ion trap. Phys. Rev. A.

[19] Colgan J (2017). Atomic structure considerations for the low-temperature opacity of Sn. High. Energy Density Phys..

[20] Torretti F (2017). Optical spectroscopy of complex open-4*d*-shell ions $${{\rm{Sn}}}^{7+}-{{\rm{Sn}}}^{10+}$$. Phys. Rev. A.

[21] Bauche J, Bauche-Arnoult C, Klapisch M (1988). Transition arrays in the spectra of ionized atoms. Adv. At. Mol. Phys..

[22] Schupp R (2019). Radiation transport and scaling of optical depth in Nd:YAG laser-produced microdroplet-tin plasma. Appl. Phys. Lett..

[23] Scott HA (2001). Cretin – a radiative transfer capability for laboratory plasmas. J. Quant. Spectrosc. Radiat. Transf..

[24] Basko MM, Novikov VG, Grushin AS (2015). On the structure of quasi-stationary laser ablation fronts in strongly radiating plasmas. Phys. Plasmas.

[25] Basko M (2016). On the maximum conversion efficiency into the 13.5-nm extreme ultraviolet emission under a steady-state laser ablation of tin microspheres. Phys. Plasmas.

[26] Su M (2017). Evolution analysis of EUV radiation from laser-produced tin plasmas based on a radiation hydrodynamics model. Sci. Rep..

[27] Fujioka S (2005). Opacity effect on extreme ultraviolet radiation from laser-produced tin plasmas. Phys. Rev. Lett..

[28] Zeng J, Gao C, Yuan J (2010). Detailed investigations on radiative opacity and emissivity of tin plasmas in the extreme-ultraviolet region. Phys. Rev. E.

[29] Magee, N. H. et al. In *AIP Conf. Proc.,* vol. 730, 168–179 (AIP, 2004)

[30] Hakel P (2006). The new Los Alamos opacity code ATOMIC. J. Quant. Spectr. Rad. Transf..

[31] Abdallah J, Clark R, Peek J, Fontes C (1994). Kinetics calculations for near Ne-like ions. J. Quant. Spectr. Rad. Transf..

[32] Fontes C (2015). The Los Alamos suite of relativistic atomic physics codes. J. Phys. B: . Mol. Opt. Phys..

[33] Cowan, R. D. *The Theory of Atomic Structure and Spectra* (University of California Press, 1981).

[34] Hakel, P. & Kilcrease, D. P. In *AIP Conf. Proc*., vol. 730, 190–199 (AIP, 2004).

[35] Kilcrease, D., Colgan, J., Hakel, P., Fontes, C. & Sherrill, M. In *Workshop on Astrophysical Opacities, vol. 515 of Astronomical Society of the Pacific Conference Series* (ASP, 2018)

[36] Sasaki A (2010). Modeling of radiative properties of Sn plasmas for extreme-ultraviolet source. J. Appl. Phys..

[37] D’Arcy R (2009). Transitions and the effects of configuration interaction in the spectra of Sn XV-Sn XVIII. Phys. Rev. A.

[38] Schupp R (2019). Efficient generation of extreme ultraviolet light from Nd:YAG-driven microdroplet-tin plasma. Phys. Rev. Appl.

[39] Meijer RA, Stodolna AS, Eikema KSE, Witte S (2017). High-energy Nd:YAG laser system with arbitrary sub-nanosecond pulse shaping capability. Opt. Lett..

[40] Goh S (2015). Fabrication and characterization of free-standing, high-line-density transmission gratings for the vacuum UV to soft X-ray range. Opt. Express.

[41] Scott, H. A. In *Modern Methods in Collisional-Radiative Modeling of Plasmas* (ed. Ralchenko, Y.), 81–104 (Springer International Publishing, Cham, 2016)

[42] Basko MM, Sasorov PV, Murakami M, Novikov VG, Grushin AS (2012). One-dimensional study of the radiation-dominated implosion of a cylindrical tungsten plasma column. Plasma Phys. Control. Fusion.

[43] Bleiner, D. et al. (eds.) *Short Wavelength Laboratory Sources* (The Royal Society of Chemistry, 2015)

[44] Legall H (2012). Compact x-ray microscope for the water window based on a high brightness laser plasma source. Opt. Express.

[45] Ohashi H (2014). Quasi-moseley’s law for strong narrow bandwidth soft x-ray sources containing higher charge-state ions. Appl. Phys. Lett..

[46] Griem HR (1963). Validity of local thermal equilibrium in plasma spectroscopy. Phys. Rev..

[47] Bauche, J., Bauche-Arnoult, C. & Peyrusse, O. *Atomic Properties in Hot Plasmas* (Springer International Publishing, 2015)

[48] Nikiforov, A. F., Novikov, V. G. & Uvarov, V. B. *Quantum-Statistical Models of Hot Dense Matter: Methods for Computation Opacity and Equation of State*, vol. 37 (Springer Science & Business Media, 2006)

[49] Basko, M. M., Maruhn, J. & Tauschwitz, A. Development of a 2D radiation-hydrodynamics code RALEF for laser plasma simulations, GSI Report 2010-1, PLASMAPHYSICS- 25 (GSI Helmholtzzentrum flur Schwerionenforschung GmbH, 2010).

[50] Kurilovich D (2018). Power-law scaling of plasma pressure on laser-ablated tin microdroplets. Phys. Plasmas.

